# Feasibility Study of an Assay Measuring Activity of Oral Cancer Protease Mixtures to Cleave Protease-Activated Receptor 2, an Oral Cancer Pain Mediator

**DOI:** 10.3390/ijms27208971

**Published:** 2026-10-09

**Authors:** Paulina D. Ramirez-Garcia, Igor Dolgalev, Sarah Zubli, Brian L. Schmidt, Donna G. Albertson

**Affiliations:** 1 Translational Research Center, NYU Dentistry, New York, NY 10010, USA; p.ramirez.garcia@uc.cl (P.D.R.-G.); sz3217@nyu.edu (S.Z.); 2 Department of Medicine, NYU Langone Health, New York, NY 10016, USA; igor.dolgalev@nyulangone.org; 3 Translational Research Center and the Pain Research Center, NYU Dentistry, New York, NY 10010, USA

**Keywords:** protease-activated receptor 2, PAR_2_, trypsin, ST14 (matriptase), MMP1, PRSS23, PAR_2_ N-terminus cleavage, oral cancer, protease activity, saliva

## Abstract

Oral cancer patients suffer pain that is patient-specific and heterogeneous at the site of the tumor. Protease-activated receptor 2 (PAR_2_), a G-protein coupled receptor and an oral cancer mediator, is activated by protease cleavage of an N-terminal ligand. Once cleaved, the receptor is retained at the plasma membrane or re-distributed (trafficking) to internal sites followed by downstream protease-specific signaling. The oral cancer tumor microenvironment (TME) is replete with protases that activate PAR_2_, potentially evoking PAR_2_ trafficking and signaling that is unique to each cancer and reflecting the protease composition of the individual TMEs. To measure PAR_2_ cleavage activity in oral cancer samples, an array of overlapping peptides spanning the PAR_2_ N-terminus was assembled. In this feasibility study, we assayed the cleavage activity of individual PAR_2_-activating proteases and mixtures of proteases modeling the oral cancer TME. Additionally, we confirmed that PAR_2_-cleaving proteases are active in oral cancer patient saliva, a sample that can be collected non-invasively to measure PAR_2_ cleavage in patients when they are experiencing pain prior to surgery. Further studies should elucidate the utility of the peptide array to measure PAR_2_-activating proteolysis in cancer samples.

## 1. Introduction

Oral cancer, a member of the group of head and neck cancers that is not associated with human papilloma virus (HPV) infection, is painful at the site of the cancer. Seventy percent of oral cancer patients self-report pain [1] on the University of California San Francisco Oral Cancer Pain Questionnaire (UCSFOCPQ), a validated oral cancer pain questionnaire [2,3]. The intensity and character of oral cancer pain vary among patients [1,4]. Oral cancer can generate severe, debilitating, function-induced pain that cannot be alleviated with opioids or other analgesics. This form of pain is hypothesized to be caused by the release of mediators in the TME that sensitize trigeminal ganglion (TG) sensory neurons. Patients with node positive (N+) oral cancers, that is, the oral cancer metastasized to the cervical lymph nodes, report greater pain in the oral cavity [1].

Protease-activated receptor 2 (PAR_2_), encoded by *F2RL1*, is a mediator of oral cancer pain and other painful syndromes [5,6,7,8,9]. PAR_2_ is a member of the family of G-protein coupled receptors (GPCRs) and is activated by proteolytic cleavage of an extracellular N-terminal tethered ligand. Trypsin and other proteases that cleave PAR_2_ at the same site (R^36^↓S^37^) lead to canonical PAR_2_ trafficking and signaling, i.e., internalization of the receptor to endosomes and Ca^2+^ signaling. Protease cleavage of PAR_2_ at different (non-canonical) sites exposes different tethered ligands that either disarm the receptor, abrogating canonical signaling, or promote protease-specific retention of PAR_2_ at the plasma membrane or trafficking of PAR_2_ to intracellular sites and signaling (biased agonism) [10]. 

Dysregulated proteases, abundant in the oral cancer TME, can both promote cancer [11] and activate PAR_2_ by cleavage at canonical and non-canonical sites. Proteases reported to produce oral cancer pain via PAR_2_ include one canonical protease, transmembrane serine protease 2 (encoded by *TMPRSS2*), and two non-canonical proteases, cathepsin S (encoded by *CTSS*) and legumain (encoded by *LGMN*) [12,13,14]. Additionally, two proteases, matrix metallopeptidase 1 (encoded by *MMP1*) and serine protease 23 (encoded by *PRSS23*) are more highly expressed in N+ cancers in patients expressing the highest levels of pain [1]. Activation of PAR_2_ by MMP1 or PRSS23 promotes trafficking of PAR_2_ to the Golgi and an increase in cAMP levels [4], whereas the receptor remains at the plasma membrane when activated by LGMN or CTSS, resulting in protease-specific signaling [14,15]. Studies in vitro demonstrate that activation of PAR_2_ by mixtures of canonical and non-canonical proteases elicit trafficking and signaling that depend on the concentrations of proteases and their relative potencies and efficacies [4]. 

These observations highlight the potential impact of the protease composition of the TME to promote PAR_2_ trafficking to multiple intracellular sites in painful cancers. Greater antagonism of PAR_2_ and other GPCRs that are endocytosed and delivered to intracellular compartments where they continue to signal is achieved by delivery of drugs to the intracellular sites of the receptors [16,17,18,19,20,21,22]. PAR_2_, however, presents a particular challenge to such an approach, because activation of PAR_2_ elicits protease-specific trafficking of PAR_2_ to different intracellular sites. Thus, strategies to deliver drugs to intracellular sites to antagonize PAR_2_ would benefit from knowledge of the potential impact of the cocktail of proteases present in the TME on PAR_2_ cleavage and the associated trafficking and signaling. To this end, we assembled an array of overlapping peptides to measure cleavage of the N-terminus of PAR_2_ that did not rely on knowledge of specific cleavage sites. Cell-based studies of individual PAR_2_-activating proteases and mixtures of proteases have demonstrated a linkage between the site of cleavage of the N-terminus and downstream signaling [4]. Therefore, we reasoned that the patterns of cleavage of the N-terminus of PAR_2_ might be developed into an assay to predict PAR_2_ trafficking and signaling. Our aim is to measure cleavage activity as a predictor of the PAR_2_ trafficking and signaling promoted by oral cancer in order to inform targeting of PAR_2_ antagonists to intracellular sites of receptor signaling. In this initial feasibility study, we investigated cleavage by individual proteases and observed that the pattern of cleavage reported by the array in response to mixtures of proteases varied with the concentration of MMP1 in the mixture. We also confirmed that PAR_2_-activating proteases are active in the saliva of oral cancer patients, supporting the utility of saliva, a non-invasively collected sample, to interrogate PAR_2_ cleavage activity in the oral cancer TME. 

## 2. Results

### 2.1. Abundance of Canonical and Non-Canonical Serine and Other Proteases Reported to Cleave PAR_2_ in Oral Cancers 

**Figure 1:**
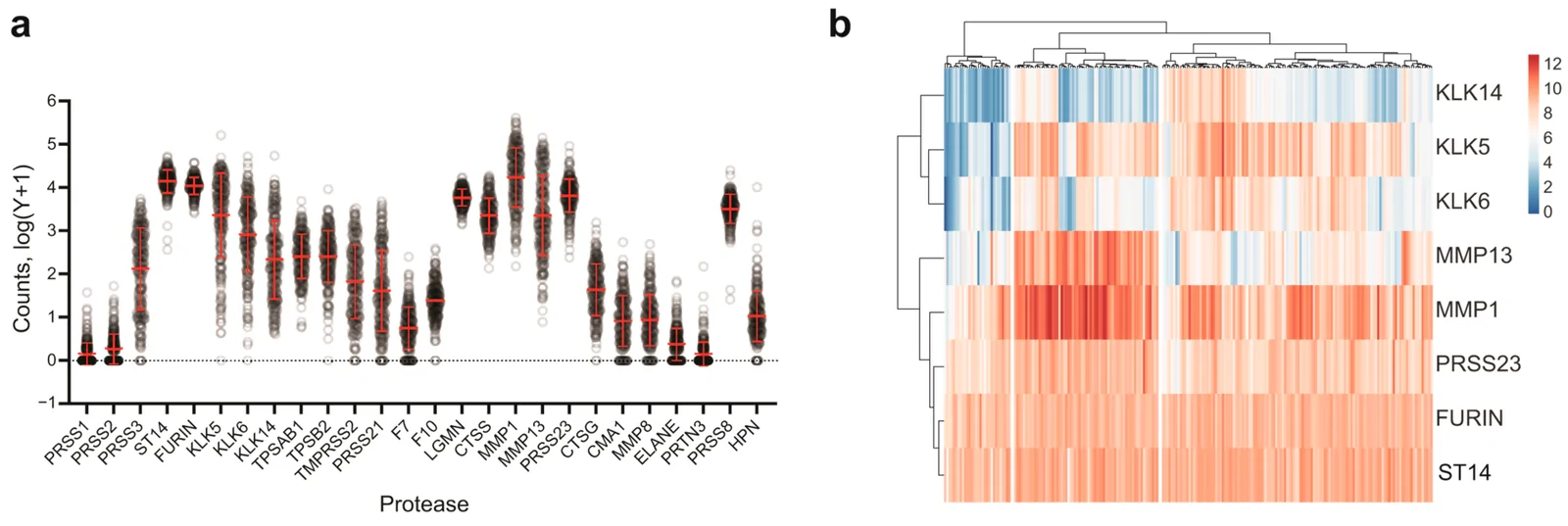
Abundance of canonical and non-canonical serine and other proteases reported to cleave PAR_2_. (**a**) Protease expression (normalized counts) in 217 bulk RNA sequenced oral cavity cancers from the TCGA PanCancer Atlas. (**b**) Hierarchical clustering of the 217 patients according to expression of the 15 more abundant PAR_2_-cleaving proteases. Normalized counts were ln(x + 1) transformed. Rows and columns were clustered by Euclidian distance and Ward linkage. No scaling or centering was applied to rows. The patients are shown in columns and proteases in rows.

To identify PAR_2_-cleaving proteases in oral cancers, we downloaded gene expression data (bulk RNA sequencing) from the TCGA PanCancer Atlas [23] and filtered the dataset for oral cavity cancers according to ICD-10 code (Figure 1a). The gene *PRSS1* encoding trypsinogen, the inactive precursor protein of trypsin, is expressed at low levels in oral cancers compared to other genes encoding canonical proteases that cleave at the same site as trypsin, such as *ST14* (matriptase), *FURIN*, kallikreins (*KLK5*, *KLK6*, *KLK14*), *F7* and *F10* (encoding coagulation factors VIIa and Xa), tryptases (*TPSAB1*, *TPSB2*) and *PRSS21* (gene encoding testisin). Other serine proteases (prostasin and HPN, encoded by *PRSS8* and *HPN*, respectively) promote canonical cleavage by activation of ST14. Abundant transcripts of non-canonical proteases included *LGMN*, *CTSS*, *MMP1*, *MMP13* and *PRSS23*.

The large number of PAR_2_-activating proteases and the heterogeneity in the cocktails of proteases present in oral cancers (Figure 1b) motivated our search for a means to assay how PAR_2_ might be activated in individual patients’ cancers. Whereas transcript levels of canonical proteases *ST14* and *FURIN* are relatively constant across samples, there are cancers with higher expression of *MMP1*, *MMP13* and *PRSS23* (Figure 1b). Thus, there is potential for different canonical and non-canonical PAR_2_ trafficking and signaling across the various oral cancer clusters, as suggested by the cell-based studies [4].

### 2.2. Cleavage by Canonical and Non-Canonical Proteases at Known PAR_2_ N-Terminus Sites Was Identified on the Peptide Array

**Figure 2:**
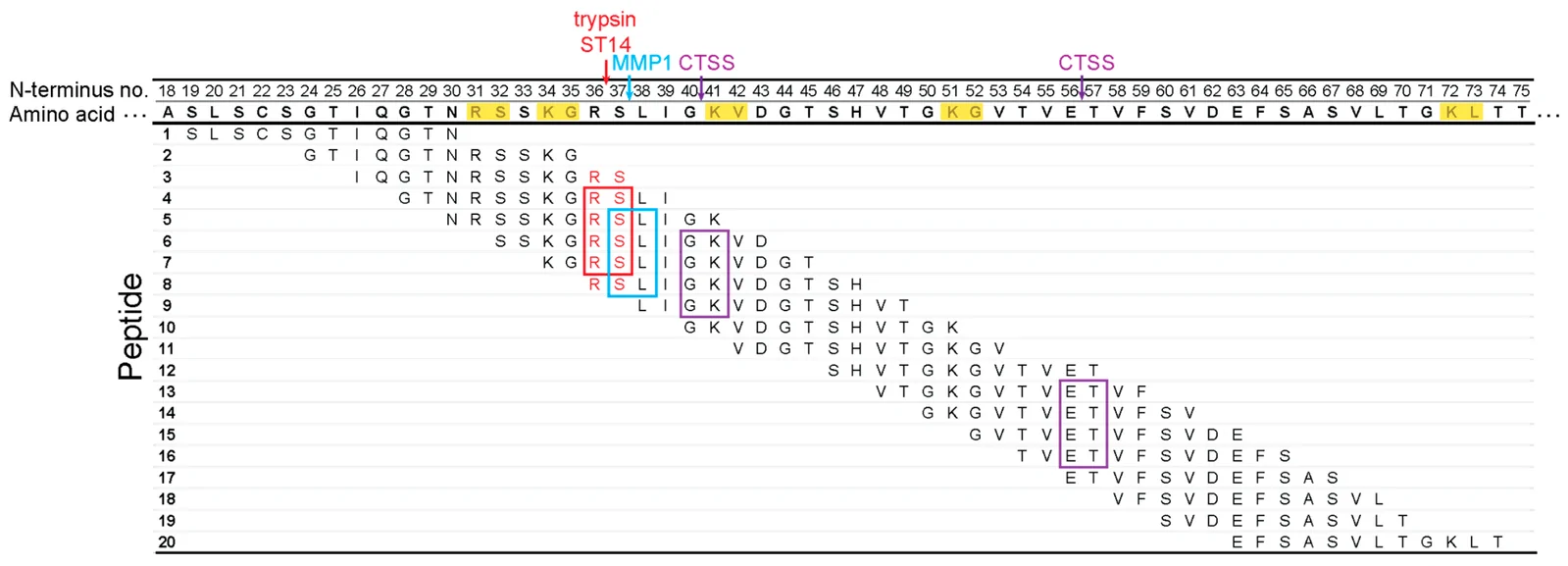
Alignment of peptides #1–20 on the PAR_2_ N-terminus amino acid sequence. Peptide sequences were shifted 2–5 amino acids along the N-terminus. Known cleavage sites for trypsin, ST14, MMP1 and CTSS contained within peptides are indicated by boxes (red = trypsin and ST14, blue = MMP1, purple = CTSS). The canonical trypsin cleavage site at R^36^↓S^37^ is indicated in red. Amino acids at additional trypsin cleavage sites are highlighted in yellow.

The peptide array comprised nineteen 12-mer and one 11-mer (#19) overlapping peptides shifted by 2 to 5 amino acids and spanning the PAR_2_ N-terminus from S^19^ to T^74^ (Figure 2). Incomplete measurements were made with peptide #1 due to insolubility and peptides #14 and #20, which were used in preliminary experiments. 

**Figure 3:**
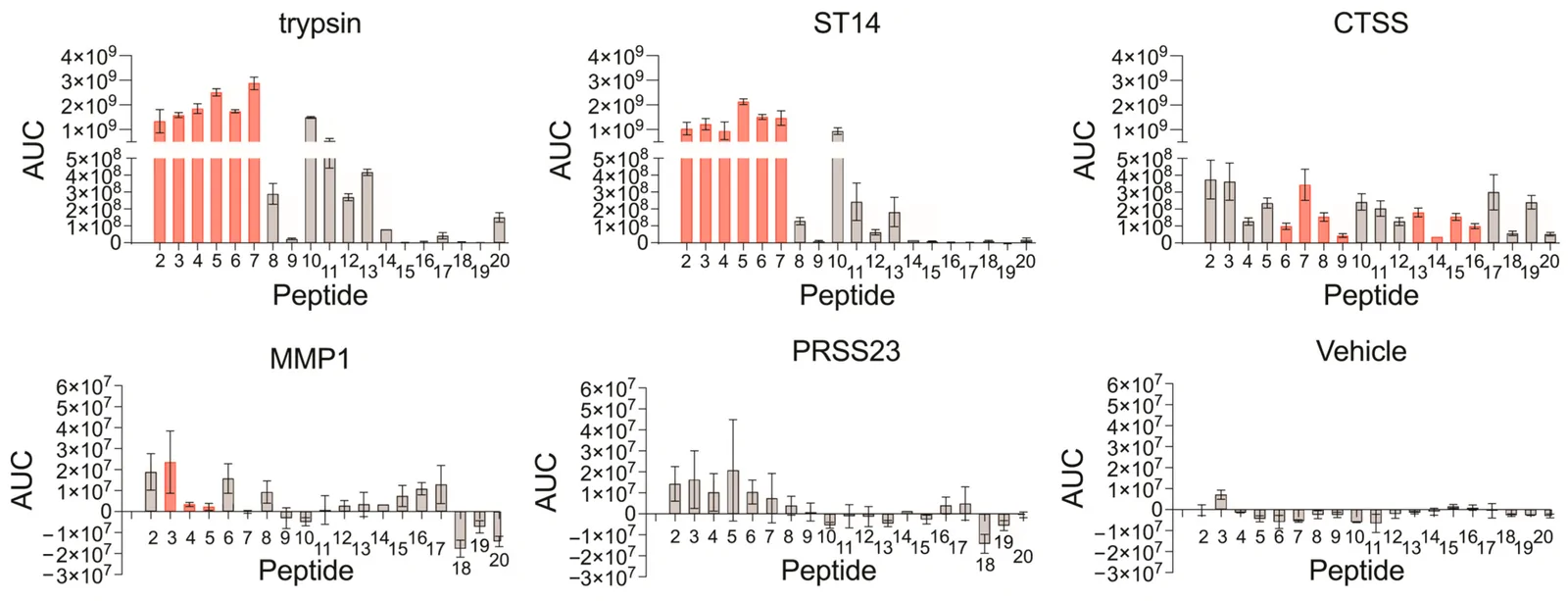
Protease cleavage at known and potentially novel sites. Shown are the net AUCs of the dose–response curves relative to the vehicle for each protease and the vehicle alone. Peptides encompassing known cleavage sites of proteases are indicated in red (trypsin and ST14 at R^36^↓S^37^, MMP1 at S^37^↓L^38^ and CTSS at G^40^↓K^41^ and E^56^↓T^57^). Maximum responses by MMP1 and PRSS23 were approximately 100-fold lower than responses at the known trypsin and ST14 cleavage sites.

Seven graded concentrations of trypsin, ST14, CTSS, MMP1 and PRSS23 ranging from 10 pM to 1 µM (1 µM, 100 nM, 10 nM, 1 nM, 100 pM, 10 pM, and 1 pM) were applied to the array (Supplementary Data Files S1–S3). The net area under the curve (AUC) of the baseline subtracted response relative to the vehicle (PBS) was calculated for each peptide at each concentration (Supplementary Figures S1–S4), and the net AUCs for these dose-response curves are shown in Figure 3. Greater median net AUCs were observed for trypsin (median = 2.89 × 10^8^, range 5.45 × 10^5^–2.87 × 10^9^), ST14 (median = 1.28 × 10^8^, range = −3.22 × 10^6^–2.13 × 10^9^) and CTSS (median = 1.54 × 10^8^, range = 3.70 × 10^7^–3.75 × 10^8^) compared to MMP1 (median = 3.25 × 10^6^, range = −1.77 × 10^7^–2.36 × 10^7^) and PRSS23 (median = 1.36 × 10^6^, range = −1.43 × 10^7^–2.07 × 10^7^) or vehicle (median = −2.44 × 10^6^, range = −6.60 × 10^6^–7.09 × 10^6^).

Maximum cleavage by the canonical proteases trypsin and ST14 was observed at peptides #2–7 and #10 (Figure 3). With the exception of peptide #6, the dose–response curves for trypsin were shifted to the left of ST14, suggesting greater potency (Supplementary Figure S1). Included within peptides #3–7 are trypsin cleavage sites at K^34^↓G^35^ and R^36^↓S^37^, previously demonstrated in a large peptide spanning the human PAR_2_ N-terminus from amino acids R^31^ to P^79^ (sites K^34^↓G^35^, R^36^↓S^37^, K^51^↓G^52^ and K^72^↓L^73^) [24]. Analysis of cleavage by the trypsin used in this study of three 30-amino acid peptides spanning the human PAR_2_ N-terminus (amino acids 21–50, 31–60 and 61–90) revealed cleavage sites at R^31^↓S^32^, K^41^↓V^42^, K^51^↓G^52^ and K^72^↓L^73^ (Supplementary Figure S5 and Supplementary Data File S4). The high cleavage activity at peptides #2–7 may be attributed to inclusion within these peptides of combinations of sites R^31^↓S^32^, R^36^↓S^37^, K^34^↓G^35^ and K^41^↓V^42^. By contrast, we lack an explanation for the cleavage of peptide #10 by trypsin and ST14, with potency and efficacy equivalent to that observed with peptides #2–7. Peptide #10, spanning amino acids 40–51 (GKVDGTSHVTGK), includes only one site (K^41^↓V^42^) within the interior of the peptide. Additionally, cleavage activity was low with overlapping peptides #9 and #11, which are expected to include the potential high activity cleavage site(s) within peptide #10. 

The MMP1 and CTSS responses at their known cleavage sites of CTSS and MMP1 were lower than observed for trypsin and ST14 at the known trypsin and ST14 sites (10- and 100-fold, respectively), consistent with the reported greater potencies and efficacies of trypsin and ST14 to activate PAR_2_ in trafficking and signaling assays [4]. In most cases, dose–response curves were not sigmoidal, which did not allow calculation of an EC_50_ for cleavage at each peptide (Supplementary Figures S1–S4). For CTSS, increases in cleavage at 1 µM were observed at all peptides, without evidence of increased potency or efficacy at the reported CTSS cleavage sites (Figure 3 and Supplementary Figure S2). A potential MMP1 cleavage site is suggested that maps within peptides #15 and #16 (amino acids 52–65) (Figure 3 and Supplementary Figure S3). A cleavage site for PRSS23 has not been reported. No site was apparent from the net AUC dose–response curves (Figure 3 and Supplementary Figure S4). 

### 2.3. Mixtures

**Figure 4:**
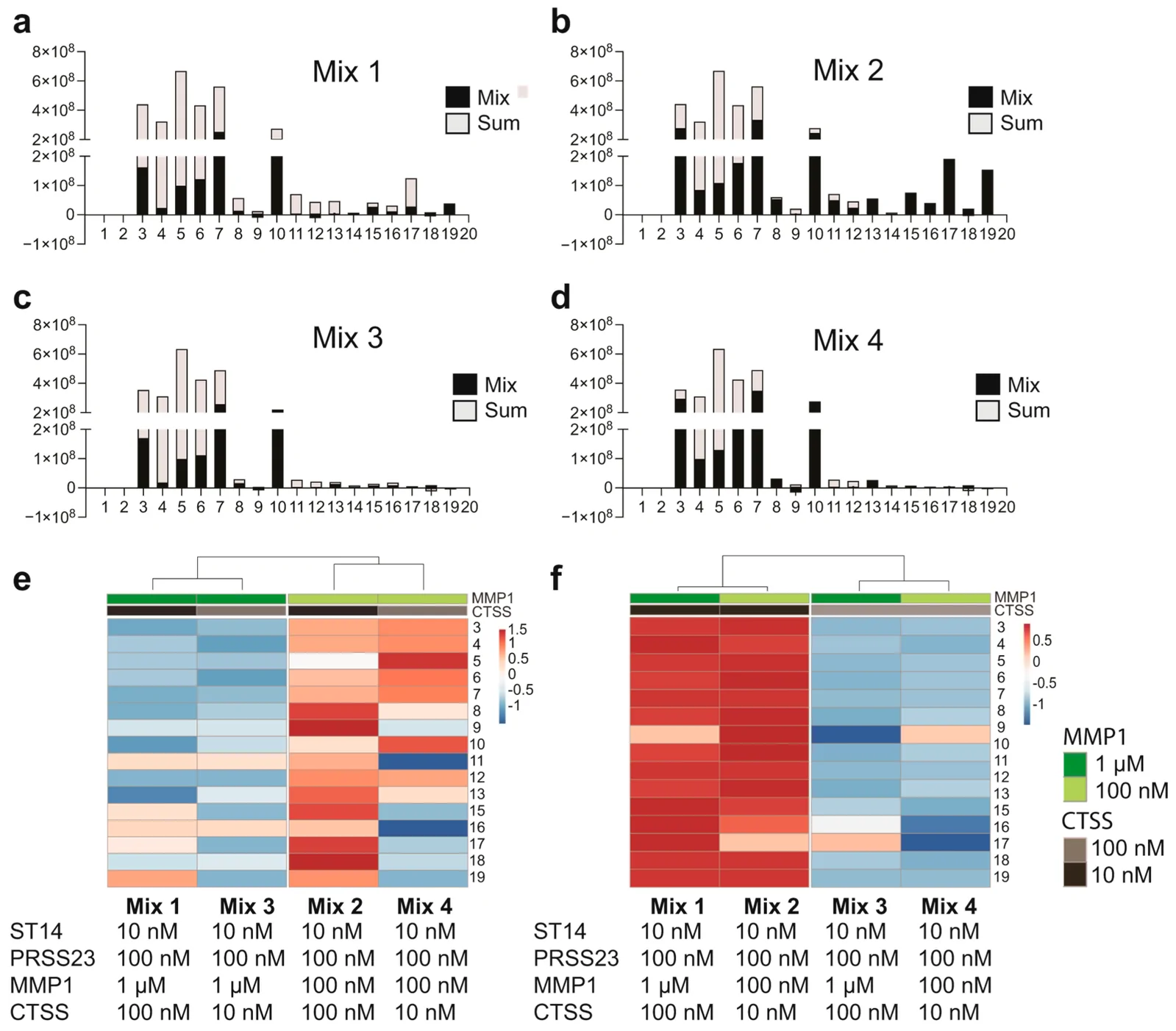
Cleavage by mixtures of proteases varies with MMP1 concentration. (**a**–**d**) Net AUC values measured at each protease for the four mixtures and the predicted responses. Hierarchical clustering of net AUC values measured for mixtures (**e**,**f**) predicted by summing the net AUC values measured for individual proteases at the concentrations in the mixtures. Measured and predicted net AUC values were ln(x + 1) transformed. Mixtures in columns were clustered by Euclidian distance and Ward linkage. Rows are centered, and unit variance scaling is applied to rows.

To begin to model cleavage by mixtures of proteases, as would be present in saliva bathing the cancer, for example, we prepared four mixtures of proteases present in oral cancers, containing ST14, CTSS, MMP1 and PRSS23 (Supplementary Data File S5). We designed the mixtures based on gene transcript levels from the TCGA study (Figure 1), our study of differentially expressed genes in oral cancers from patients reporting high levels of pain [1], which found MMP1 transcript levels to be elevated in association with pain, and recent cell-based studies demonstrating the impact of elevated levels of MMP1 in protease mixtures on PAR_2_ trafficking and signaling [4]. We note, however, that gene transcript levels are not expected to be an accurate measure of protease activity due to post-translational modifications and interactions with activators and inhibitors. All mixtures of recombinant proteases contained ST14 at 10 nM and PRSS23 at 100 nM and either 1 µM or 100 nM MMP1 together with CTSS at 100 nM or 10 nM. In cell-based studies, we previously observed that PAR_2_ trafficking and signaling evoked by mixtures of proteases could be explained by summing the activities of the individual proteases in the mixtures [4]. Therefore, we compared the measured responses of mixtures with the sum of the responses of the individual proteases at the given concentrations. In most cases, the response of the mixtures was less than predicted by the sum of the individual proteases (Figure 4a–d). Hierarchical clustering revealed that the measured net AUCs for mixtures cluster by MMP1 concentration in the mixture, while predicted values cluster by CTSS concentration (Figure 4e,f). The responses were lower with mixtures with 1 µM MMP1. Further studies will be needed to determine whether reduced cleavage at the peptides spanning the canonical site could be attributed to MMP1 activity disarming the receptor [25].

### 2.4. PAR_2_-Cleaving Proteases Are Active in Oral Cancer Patient Saliva

Saliva bathes cancers in the oral cavity and is a demonstrated source of oral cancer biomarkers [26]. A number of PAR_2_-activating proteases have been reported to be enriched in saliva from oral cancer patients [27], and studies have investigated MMP1 and MMP13 levels as biomarkers of oral cancer [28,29]. Saliva, sampled non-invasively, could have utility for assaying PAR_2_ activation in patients prior to surgery. 

**Figure 5:**
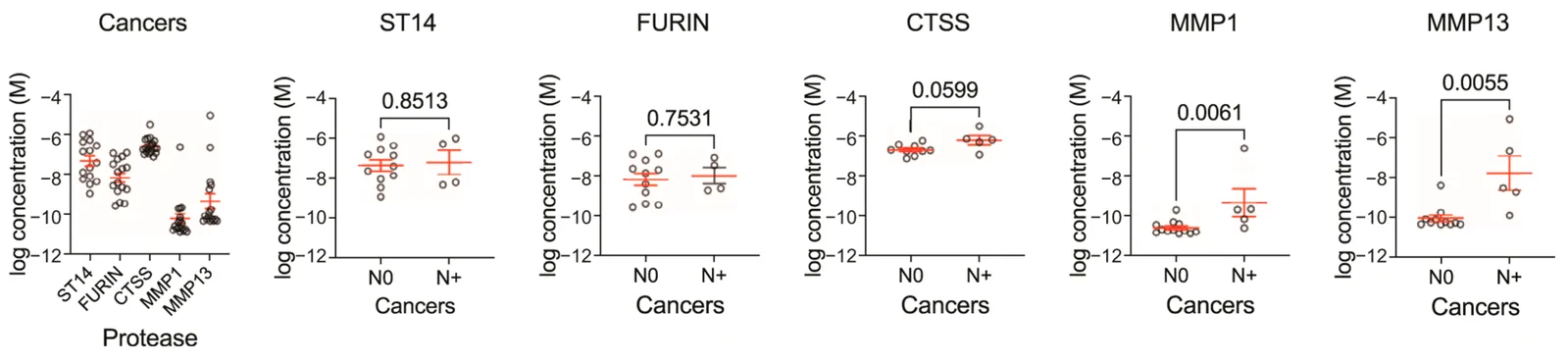
MMP1 and MMP13 activity is higher in saliva from oral cancer patients with N+ cancers. Shown are expression levels of active proteases in all cancers and for individual proteases according to nodal status of the patients’ cancers. The mean ± SEM log concentration (M) is indicated in red, and data were analyzed by a Mann–Whitney U test. For MMP1, the Mann–Whitney U test showed a significant difference between the activity in saliva from N0 (−10.28, n = 12) and N+ (−9.693, n = 5) patients, U = 5, *p* = 0.0061. The 95.15% confidence interval for the median difference is [0.1563 to 3.069]. For MMP13, the Mann–Whitney U test showed a significant difference between the activity in saliva from N0 (−10.73, n = 11) and N+ (−8.530, n = 5) patients, U = 4, *p* = 0.0055. The 96.20% confidence interval for the median difference is [0.4385 to 4.712].

To confirm that saliva from oral cancer patients would be a suitable biofluid to measure PAR_2_ cleavage activity, protease activity in saliva was measured using commercial activity kits (not PAR_2_ specific cleavage). The activity of individual proteases (ST14, FURIN, CTSS, MMP1 and MMP13) was measured in saliva samples from oral cancer patients (n = 15–18). Active protease concentrations were determined by comparison to activity of graded concentrations of recombinant proteases (Figure 5 and Supplementary Data File S6). The highest expression (log concentration, mean ± SEM) was observed for CTSS (−6.60 M ± 0.09), followed by ST14 (−7.33 M ± 0.25), FURIN (−8.16 M ± 0.23), MMP13 (−9.35 M ± 0.39), and MMP1 (−10.24 M ± 0.25). 

Since MMP13 expression levels [30] and MMP1 expression and activity [31,32] levels have been reported to be elevated in saliva from oral cancer patients with nodal disease, we asked whether the activity of the tested proteases varied with nodal status. In this exploratory study, higher activity of MMP1 and MMP13 was observed in the saliva from oral cancer patients with N+ versus node negative (N0) cancers (Figure 5). These observations on protease activity are consistent with studies reporting the presence of CTSS, MMP1 and MMP13 protein in saliva from oral cancer patients [27]. Additionally, they suggest that future studies could demonstrate a potential utility of protease activities as oral cancer biomarkers.

## 3. Discussion

We assembled the peptide array as a predictor of the biased agonism promoted by individual oral cancers. Maximal responses on the array were recorded at peptides spanning the known trypsin and ST14 cleavage sites, consistent with the high potency and efficacy of these proteases to cleave and activate PAR_2_. While the cleavage sites for CTSS, MMP1 and PRSS23 were not clearly delineated using the array, the potential impact of these less potent proteases on canonical cleavage, for example, was revealed by the mixture experiments. Earlier, cell-based studies of PAR_2_ trafficking and signaling evoked by two mixtures of equal concentrations of ST14, CTSS, PRSS23 and MMP1 found that trafficking and signaling associated with canonical cleavage dominated at low protease concentrations, whereas with a high concentration mixture, trafficking and signaling associated with the proteases with lower potency and efficacy prevailed [4]. In the cell-based study, trafficking and signaling were predicted by summing the activities of individual proteases. Here, protease mixtures, prepared to model differences in the relative concentrations of proteases in the TME, revealed the impact of higher MMP1 concentrations on canonical cleavage. Summing the cleavage responses at individual proteases did not predict behavior of the mixture. Future studies with combinations of recombinant proteases and biological samples will be needed to better understand the clinical impact of protease mixtures on PAR_2_ activation as measured in different assays.

The peptide array, assembled as a predictor of the biased agonism promoted by individual oral cancers, is a novel assay. By design, it is agnostic with respect to both the protease composition of the TME and the sites of cleavage at the PAR_2_ N-terminus. Activation of PAR_2_ promotes the pathogenesis, including inflammation and tissue destruction, of periodontitis [33], rheumatoid and osteoarthritis [9] and inflammatory bowel diseases and irritable bowel syndrome [34,35]. The prevalence of these diseases has motivated studies of the proteases and protease activities in the involved tissues [36,37,38,39]. Assays have relied on knowledge of candidate proteases and their cleavage characteristics to design probes to assay the activity of individual proteases or classes of proteases, but not PAR_2_-specific activities. One commercial assay, however, specifically monitors production of canonical PAR_2_ cleavage [40].

The prevalence and intensity of pain experienced by oral cancer patients prior to surgery motivated evaluation of saliva as a surrogate oral cancer biofluid for measuring PAR_2_ cleavage activity in the cancers. Preoperative pain in oral cancer patients is severe and notoriously difficult to manage prior to surgical resection, which remains the primary definitive cure for the cancer and the associated pain [41]. Currently, clinicians rely on systemic opioids, which frequently induce constipation, lethargy, and confusion—severely compromising a patient’s cognitive clarity and well-being during critical preoperative discussions and appointments. We note that, in addition to saliva, samples suitable for measuring protease cleavage activity in disease include tumor interstitial fluid from cancer tissue [42,43], gingival crevicular fluid or subgingival plaque (periodontitis) [44,45], feces (inflammatory bowel diseases and irritable bowel syndrome) [36,37] and synovial fluid (arthritis) [46,47]. PAR_2_ modulators [48] have entered clinical trials. Targeting PAR_2_ in endosomes improves analgesia in preclinical studies of oral cancer and diseases of the colon [16,22]. Knowledge of protease activity at the PAR_2_ N-terminus in patients could allow clinicians to select drugs appropriately targeted to the site of intracellular PAR_2_ signaling and improve analgesia.

There are limitations to the assay design and this feasibility study. The assay uses synthetic peptides, which do not recapitulate the conformation and protease modifications of the PAR_2_ N-terminus that would be present in vivo, such as glycosylation, demonstrated to modify cleavage sensitivity [49,50]. Future studies to optimize and validate the assay will investigate optimal peptide lengths and overlaps and could include peptides with mutated cleavage sites as controls, for example. Only a limited number of proteases and protease concentrations were studied due to costs. Additional PAR_2_-cleaving proteases will need to be studied and assay parameters (e.g., sensitivity, specificity, reproducibility) investigated in defined protease mixtures and clinical samples. Studies will also be required to define the downstream signaling associated with observed patterns of cleavage.

## 4. Materials and Methods

### 4.1. Proteases and Protease Activity Kits

Recombinant human proteases were purchased: MMP1 (Cat.# 420-01, PeproTech^®^, ThermoFisher Scientific, Cranbury, NJ 08512, USA), CTSS (Cat.# 219343, MilliporeSigma, Burlington, MA 01803, USA), PRSS23 (Cat.# RPU53309, Biomatik, Kitchener, ON N2C 1N6, Canada) and ST14 (Matriptase/ST14, Cat.# 3946-SEB, Bio-techne, Minneapolis, MN 55413, USA). Trypsin, obtained from porcine pancreas, was purchased from MilliporeSigma (Cat. #T0303). Proteases were resuspended in PBS (11 µM) and stored at −80 °C. Protease activity kits purchased from AnaSpec, Inc. (Freemont, CA 94555, USA) included SensoLyte^®^ Rh110 Matriptase Activity Assay Kit Fluorimetric (Cat.# AS-72241), SensoLyte^®^ Rh110 Furin Activity Assay Kit Fluorimetric (Cat.# AS-72256), SensoLyte^®^ 520 Cathepsin S kit (Cat.# AS-72099), SensoLyte^®^ Plus 520 MMP-1 Assay Kit Fluorimetric and Enhanced Selectivity (Cat.# AS-72012) and SensoLyte^®^ Plus 520 MMP-13 Assay Kit Fluorimetric and Enhanced Selectivity (Cat.# AS-72019).

### 4.2. Protease Transcript Expression Levels in Oral Cancers

Bulk RNA sequencing data for Head and Neck Squamous Cell Carcinoma (TCGA PanCancer Atlas) [23] were obtained from the UCSC Xena GDC Hub [51]. STAR (version 2.7.5c) [52] gene counts aligned to the GRCh38 reference genome (distributed as log2(count + 1)), the GENCODE v36 gene annotation probe map, and the clinical table were downloaded. Ensembl gene identifiers were mapped to HGNC symbols using the GENCODE v36 probe map. Expression values were returned to the linear count scale (2^x^ − 1). Cases were filtered for oral cavity cancers (ICD-10 codes C02.1, C02.2, C02.9, C03.0, C03.1, C03.9, C04.0, C05.0, C05.9, C06.0, C06.2), and 217 oral cavity primary squamous carcinoma samples were selected. Counts were imported into the DESeq2 (version 1.34.0) [53] R package (version 4.1.2). Genes with a count of at least 10 in more than one sample were retained. Normalized counts were exported for downstream analysis.

### 4.3. Peptide Array 

Nineteen 12-mer peptides and one 11-mer peptide spanning amino acids 19–74 of the PAR_2_ N-terminus were designed with a shift of 2–5 aa between peptides (Table 1) and synthesized by JPT Peptide Technologies, GmbH (Berlin, Germany) with the fluorophore (7-Methoxycoumarin-4-yl)acetyl (Mca) and the quencher 2,4-dinitrophenyl (Dnp) incorporated at the N- and C-termini, respectively. These peptides, internally quenched when intact, fluoresce after cleavage and subsequent removal of the quencher (Dnp). The peptides were resuspended in 100 µL of either water or DMSO, followed by water (DMSO:Water 20:80), to make stocks of 5 µg/µL. Peptides were stored at −80 °C.

To measure cleavage, peptides diluted in PBS (#1-20, 100 µL, 0.05 µg/µL) were added to 96-well black plates (Nunc Cat.# 12-566-09, ThermoFisher, Fair Lawn, NJ 07410, USA), and basal levels of Mca fluorescence were measured at 325/392 nm (excitation/emission) for 5 cycles, every 60 s, using a CLARIOstar Plus (BMG Labtech, Ortenberg, Germany). After 5 cycles, 10 µL of graded concentrations of individual proteases (trypsin, ST14, CTSS, PRSS23, and MMP1) were added to the peptides, and fluorescence was measured over 55 additional cycles. 

### 4.4. Cleavage of Human PAR_2_ by Trypsin

Three peptides spanning PAR_2_ amino acids 21–50 (Peptide 1, SCSGTIQGTNRSSKGRSLIGKVDGTSHVTG), 31–60 (Peptide 2, RSSKGRSLIGKVDGTSHVTGKGVTVETVFS) and 61–90 (Peptide 3, VDEFSASVLTGKLTTVFLPIVYTIVFVVGL) were synthesized by Biomatik (Kitchener, ON, Canada) and dissolved at 400 µM in water (Peptides 1 and 2) or DMSO (Peptide 3). Peptides (100 µM) were incubated with a vehicle (HBSS, Hank’s Balanced Salt Solution, pH 7.4) or 10 µM trypsin in HBSS for 45 min at 37 °C. The reaction (40 µL total volume) was quenched with an equal volume (40 µL) of 50% acetonitrile and 0.1% trifluoroacetic acid in water. Mass spectrometry (MS) was performed by the NYU Langone Health Proteomics Laboratory (RRID: SCR_017926). Samples were diluted 1:25 with 0.5% acetic acid. Six µL samples (~12 pmoles) were loaded on an Evosep One liquid chromatography system (Evossep Biosystems, DK-5230 Odense M, Denmark) coupled to a Thermo Scientific Q Exactive HF-X mass spectrometer (ThermoFisher Scientific, Fair Lawn, NJ 07410, USA) and eluted with a 44 min (Peptide 1 and 2 samples) or 11 min high-organic (Peptide 3 sample) gradient (Solvent A: Water, 0.1% formic acid; Solvent B: Acetonitrile, 0.1% formic acid). High-resolution full MS spectra were acquired with a resolution of 45,000 and an AGC target of 3 × 10^6^, with a maximum ion time of 45 ms and scan range of 400 to 1500 *m*/*z*. Following each full MS, twenty data-dependent high-resolution HCD MS/MS spectra were acquired. All MS/MS spectra were collected using the following instrument parameters: resolution of 15,000, AGC target of 1 × 10^5^, maximum ion time of 120 ms, one microscan, 2 *m*/*z* isolation window, fixed first mass of 110 *m*/*z*, and NCE of 27. MS/MS spectra were analyzed using Byonic software (version v5.4.10, Protein Metrics Inc., Boston, MA 02110, USA) [54].

### 4.5. Saliva Collection and Protease Activity Measurements

Oral cancer patients were enrolled at the New York University Oral Cancer Center (Table 2). All subjects gave written informed consent. Cancer patients were included if they were receiving curative treatment for an oral cavity cancer and they had not received chemotherapy or radiation treatment for a prior cancer. The study was carried out in accordance with the Code of Federal Regulations on the Protection of Human Subjects (45 CFR Part 46), the National Institutes of Health requirements for human subject research and institutional research policies and procedures of the Institutional Review Board at New York University Langone Health. The Committee on Human Research at NYU Langone Health approved this study (i10-01261_MOD103, 27 July 2026).

Unstimulated saliva was collected by instructing subjects to passively drool into a collection tube. Saliva samples were centrifuged at 10,000× *g* for 10 min at 4 °C to remove debris, and 500 µL aliquots of the clear supernatant were stored frozen at −80 °C until used. Protease activity was measured for graded concentrations of recombinant proteases (Supplementary Data File S6) and in 100 µL saliva samples (20 or 50 µL diluted 1:5 or 1:2 in PBS for ST14, CTSS, and FURIN or 100 µL for MMP1 and MMP13; Supplementary Data File S6) using a CLARIOstar Plus with fluorescence excitation/emission at 490/520.

### 4.6. Data Analysis

Data were analyzed using GraphPad Prism Software (version 11.0.2). Hierarchical clustering was performed using ClustVis [55]. The peptide array data were analyzed by first subtracting the baseline (mean of the first 5 readings prior to protease addition) from the raw values and subsequently from the vehicle (1–5 replicates). For the dose–response studies, three independent peptide array experiments were performed with graded concentrations of the proteases, yielding two to three measurements per dose. Single measurements were made for the array mixture experiments and patient saliva activity assays.

## Figures and Tables

**Table 1:** Peptides spanning amino acids 19–74 of the PAR_2_ NH_2_ N-terminus.

Peptide #	Aa Positions	Aa Shift	Sequence	Solvent
1	19–30	5	Mca-SLSCSGTIQGTN-Lys(Dnp)-NH_2_	Water
2	24–35	2	Mca-GTIQGTNRSSKG-Lys(Dnp)-NH_2_	Water
3	26–37	2	Mca-IQGTNRSSKGRS-Lys(Dnp)-NH_2_	Water
4	28–39	2	Mca-GTNRSSKGRSLI-Lys(Dnp)-NH_2_	Water
5	30–41	2	Mca-NRSSKGRSLIGK-Lys(Dnp)-NH_2_	Water
6	32–43	2	Mca-SSKGRSLIGKVD-Lys(Dnp)-NH_2_	Water
7	34–45	2	Mca-KGRSLIGKVDGT-Lys(Dnp)-NH_2_	Water
8	36–47	2	Mca-RSLIGKVDGTSH-Lys(Dnp)-NH_2_	Water
9	38–49	2	Mca-LIGKVDGTSHVT-Lys(Dnp)-NH_2_	Water
10	40–51	2	Mca-GKVDGTSHVTGK-Lys(Dnp)-NH_2_	Water
11	42–53	2	Mca-VDGTSHVTGKGV-Lys(Dnp)-NH_2_	Water
12	46–57	4	Mca-SHVTGKGVTVET-Lys(Dnp)-NH_2_	Water
13	48–59	2	Mca-VTGKGVTVETVF-Lys(Dnp)-NH_2_	20:80 DMSO:Water
14	50–61	2	Mca-GKGVTVETVFSV-Lys(Dnp)-NH_2_	20:80 DMSO:Water
15	52–63	2	Mca-GVTVETVFSVDE-Lys(Dnp)-NH_2_	20:80 DMSO:Water
16	54–65	2	Mca-TVETVFSVDEFS-Lys(Dnp)-NH_2_	20:80 DMSO:Water
17	56–67	2	Mca-ETVFSVDEFSAS-Lys(Dnp)-NH_2_	20:80 DMSO:Water
18	58–69	2	Mca-VFSVDEFSASVL-Lys(Dnp)-NH_2_	20:80 DMSO:Water
19	60–70	2	Mca-SVDEFSASVLT-Lys(Dnp)-NH_2_	20:80 DMSO:Water
20	63–74	3	Mca-EFSASVLTGKLT-Lys(Dnp)-NH_2_	20:80 DMSO:Water

**Table 2:** Saliva patient characteristics.

		Number
Sex	Female	9
	Male	9
Age	<60	8
	>60	10
Site	Buccal mucosa	1
	Floor of mouth	1
	Gingiva	5
	Tongue	10
	Multiple sites (ventral tongue, floor of mouth)	1
T stage *	T1	8
	T2	9
	T4a	1
Nodal status *	N0	13
	N+	5
Tobacco use	Never, non-smoker	16
	Smoker	2
Alcohol use	Never, non-drinker	10
	Current	8

* Clinical or pathological staging.

## Data Availability

All data generated in this study are available in the main text or the Supplementary Data Tables. Head and Neck Squamous Cell Carcinoma (TCGA PanCancer Atlas) data are available from the NCI Genomic Data Commons.

## Supplementary Materials

The following supporting information can be downloaded at https://www.mdpi.com/article/10.3390/ijms27208971/s1.

## Abbreviations

The following abbreviations are used in this manuscript:

PAR_2_	Protease-activated receptor 2
TME	Tumor microenvironment
HPV	Human papilloma virus
UCSFOCPQ	University of California San Francisco Oral Cancer Pain Questionnaire
N+	Node positive
*F2RL1*	Gene encoding PAR_2_, F2R like trypsin receptor 1
GPCR	G-protein coupled receptor
R^36^↓S^37^	Denotes cleavage at PAR_2_ N-terminus between amino acids #36 (arginine) and #37 (serine)
*TMPRSS2*	Transmembrane serine protease 2
*CTSS*	Gene encoding cathepsin S
*LGMN*	Gene encoding legumain
MMP1	Matrix metallopeptidase
*PRSS23*	Serine protease 23
cAMP	Cyclic adenosine monophosphate
TCGA	The Cancer Genome Atlas
ICD-10	10th revision of the World Health Organization’s International Classification of Diseases
UCSC	University of California Santa Cruz
GDC	Genomic Data Commons
STAR	Spliced Transcripts Alignment to a Reference software
GRCh38	Genome Reference Consortium human reference genome assembly 38
HUGO	Human Genome Organization
HGNC	HUGO Gene Nomenclature Committee
*PRSS1*	Gene encoding trypsinogen, the inactive precursor protein of trypsin
*ST14*	ST14 transmembrane serine protease matriptase, gene encoding matriptase
*FURIN*	Furin, paired basic amino acid cleaving enzyme
*KLK5*	Gene encoding kallikrein related peptidase 5
*KLK6*	Gene encoding kallikrein related peptidase 6
*KLK14*	Gene encoding kallikrein related peptidase 14
*F7*	Gene encoding coagulation factor VIIa
*F10*	Gene encoding coagulation factor Xa
*TPSAB1*	Gene encoding tryptase alpha/beta 1
*TPSB2*	Gene encoding tryptase beta 2
*PRSS21*	Gene encoding serine protease 21 (testisin)
*PRSS8*	Gene encoding serine protease 8 (CAP1 or prostasin)
HPN	Gene encoding hepsin
*MMP13*	Gene encoding matrix metallopeptidase 13
S^19^ to T^74^	PAR_2_ N-terminus amino acids #19 (serine) to #74 (threonine)
AUC	Area under the curve
S^37^↓L^38^	Denotes cleavage at PAR_2_ N-terminus between amino acids #37 (serine) and #38 (leucine)
G^40^↓K^41^	Denotes cleavage at PAR_2_ N-terminus between amino acids #40 (glycine) and #41 (lysine)
E^56^↓T^57^	Denotes cleavage at PAR_2_ N-terminus between amino acids #56 (glutamic acid) and #57 (threonine)
ANOVA	Analysis of variance
PBS	Phosphate-buffered saline
Mca	(7-Methoxycoumarin-4-yl)acetyl, fluorescent donor group for Förster Resonance Energy Transfer assay
Dnp	2,4-dinitrophenyl, quencher group for Förster Resonance Energy Transfer assay
DMSO	Dimethyl sulfoxide
*g*	Gravity
µM	Micromolar
nM	Nanomolar
pM	Picomolar
MS	Mass spectrometry
AGC	Automatic gain control
ms	Milliseconds
*m*/*z*	Mass-to-charge ratio
MS/MS	Tandem mass spectrometry
HCD	Higher-energy collisional dissociation
NCE	Normalized collision energy
