# Supplementary material for: Feasibility Study of an Assay Measuring Activity of Oral Cancer Protease Mixtures to Cleave Protease-Activated Receptor 2, an Oral Cancer Pain Mediator
Source: Int J Mol Sci. 2026 Oct 9;27(20):8971. doi: 10.3390/ijms27208971 (PMC13704349; doi:10.3390/ijms27208971)
Supplement: Supplementary file 1 [file ijms-27-08971-s001.zip › Supplementary figures and Data files/Supplementary Figures S1-S5.pdf]

Figure S1. ST14 and trypsin. Net AUC of the dose-response at each peptide for ST14 and trypsin is plotted vs. protease concentration.

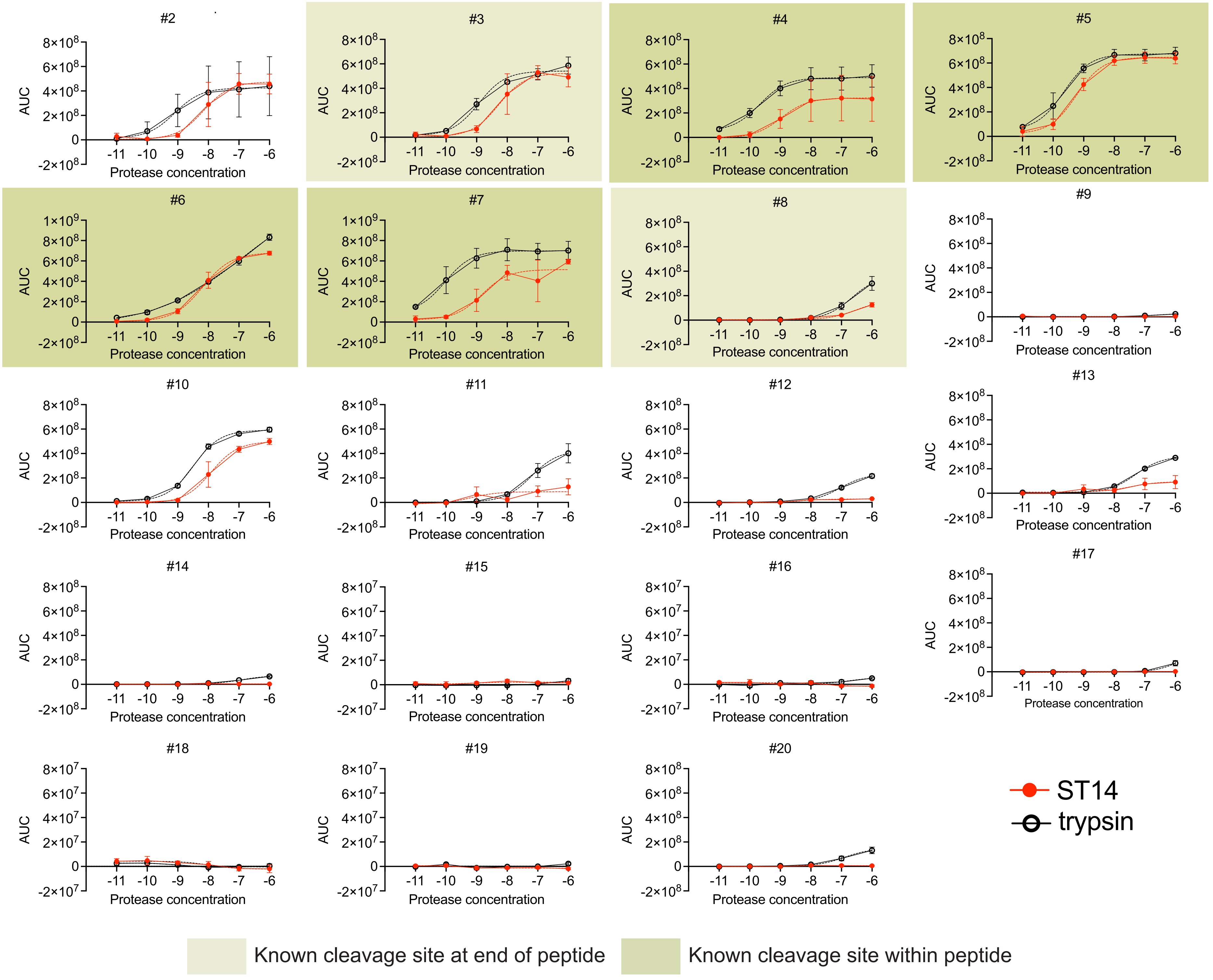

Figure S2. **CTSS**. Net AUC of the dose-response at each peptide for CTSS is plotted vs. protease concentration.

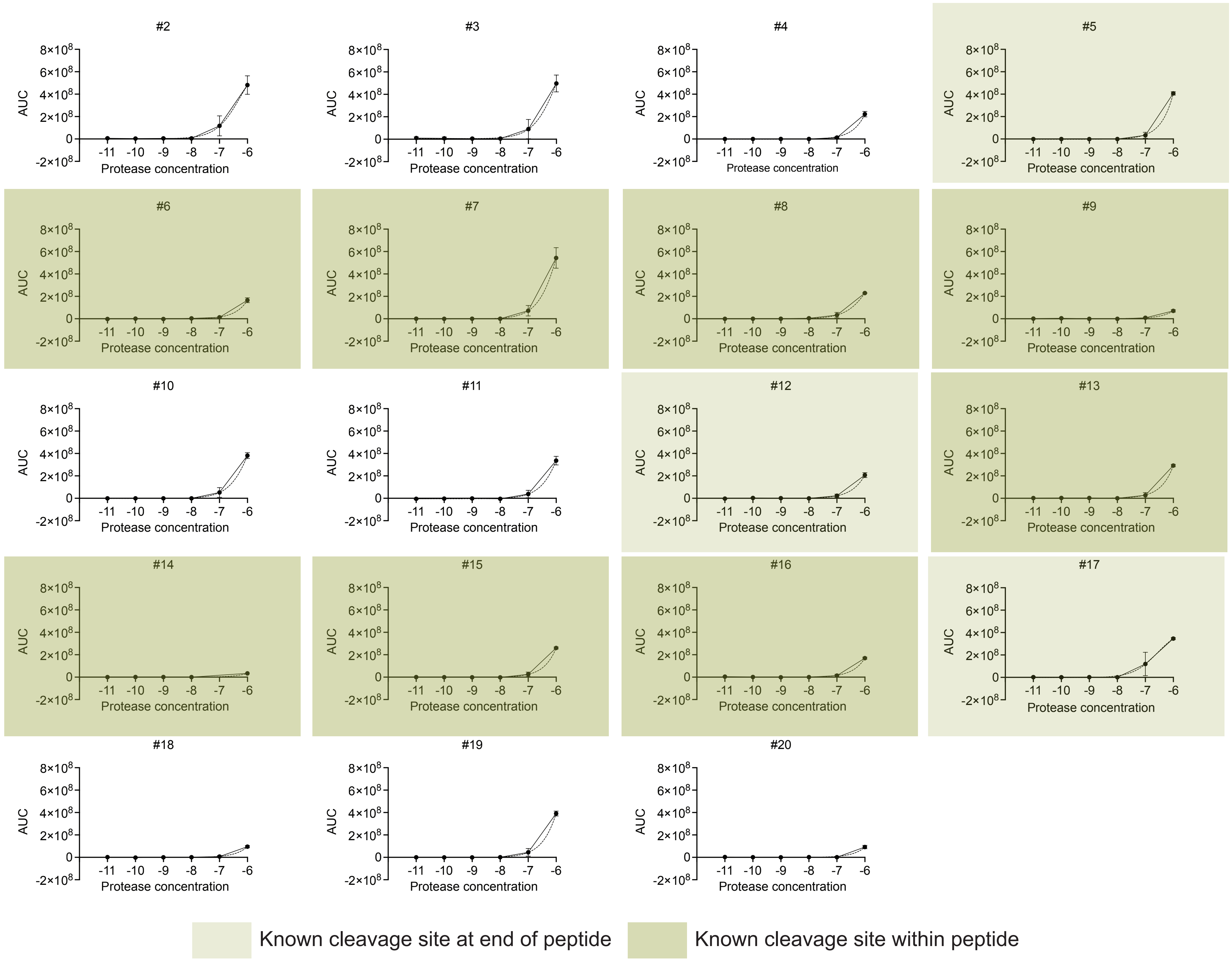

Figure S3. **MMP1**. Net AUC of the dose-response at each peptide for MMP1 is plotted vs. protease concentration.

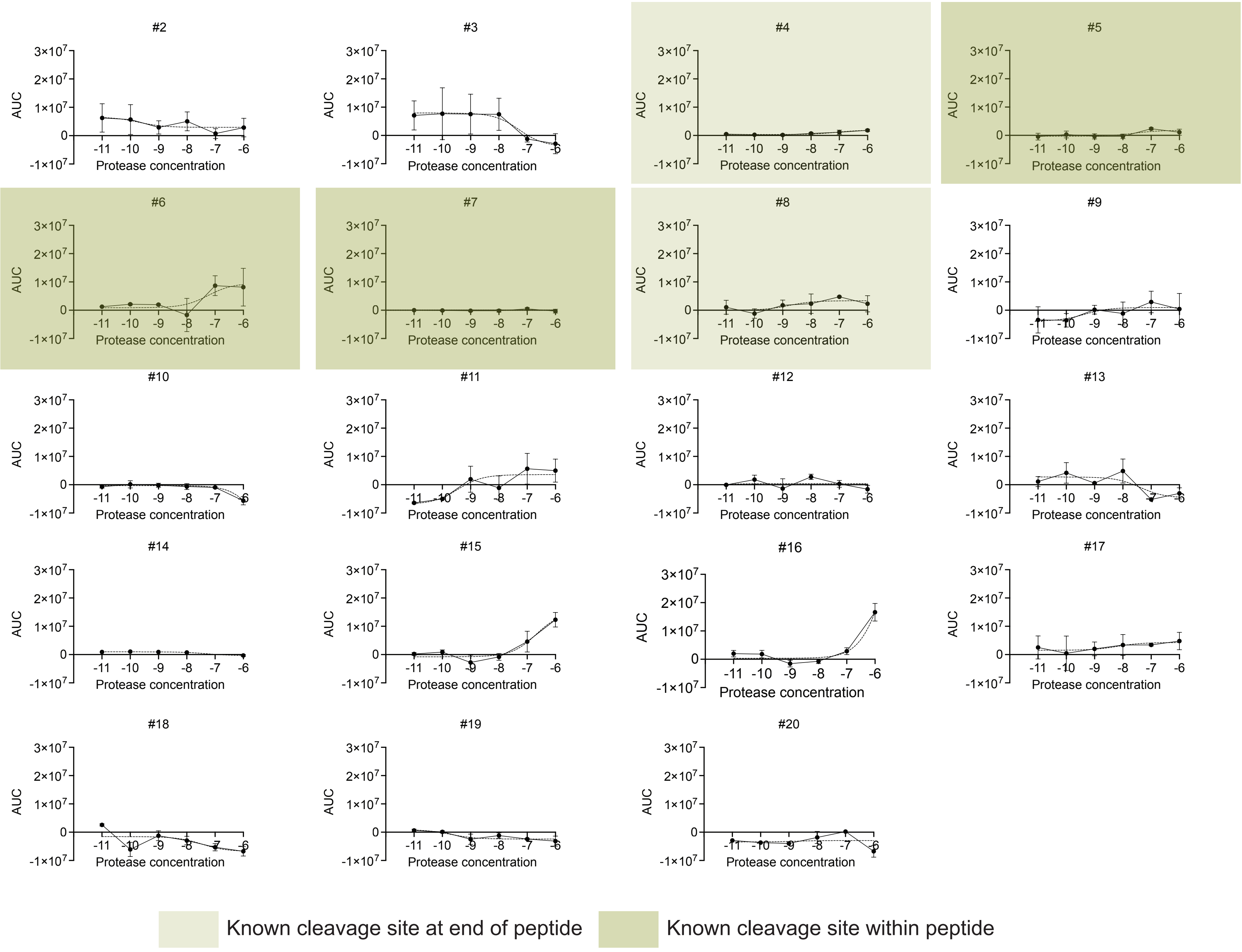

Figure S4. **PRSS23**. Net AUC of the dose-response at each peptide for PRSS23 is plotted vs. protease concentration.

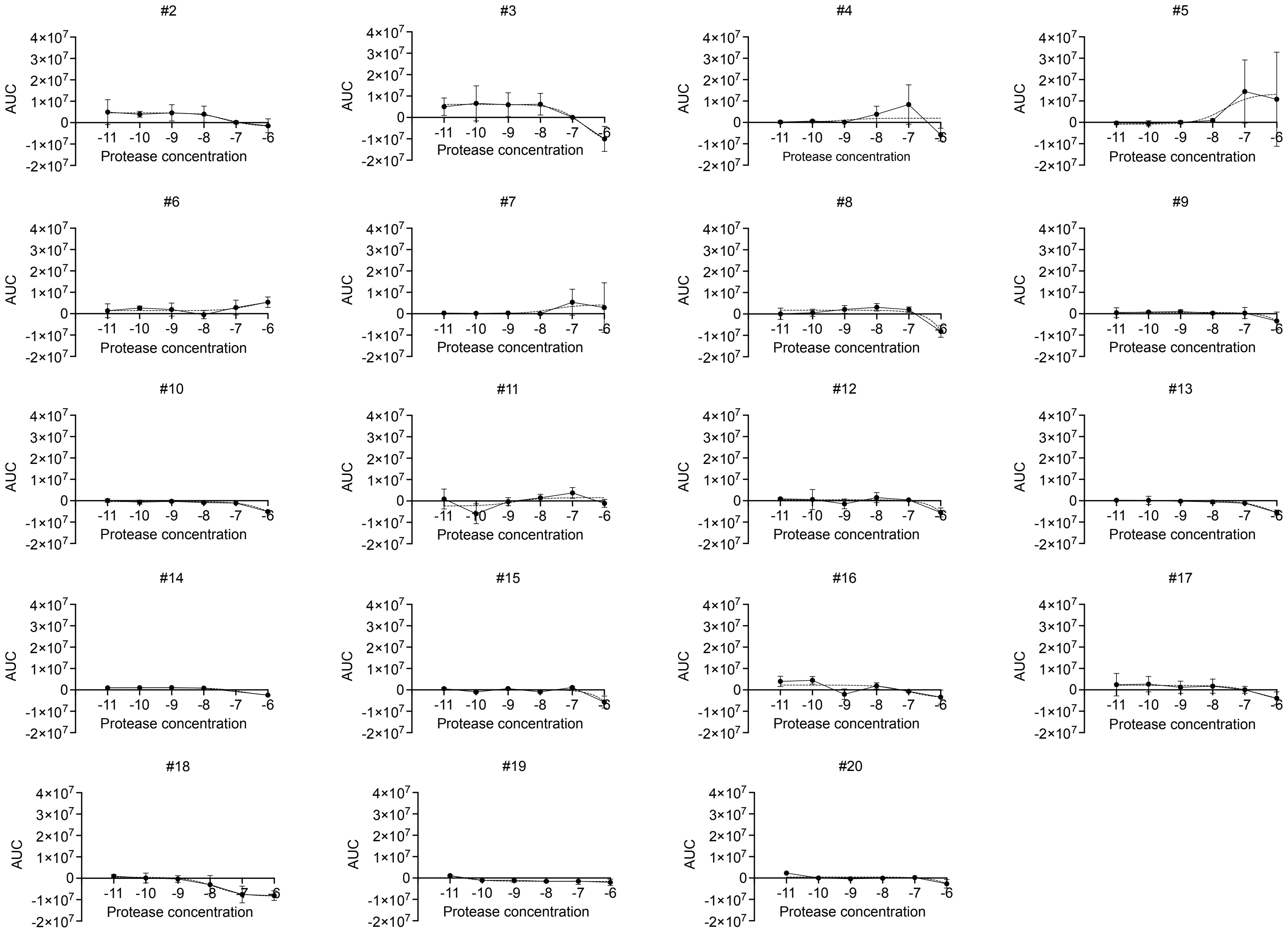

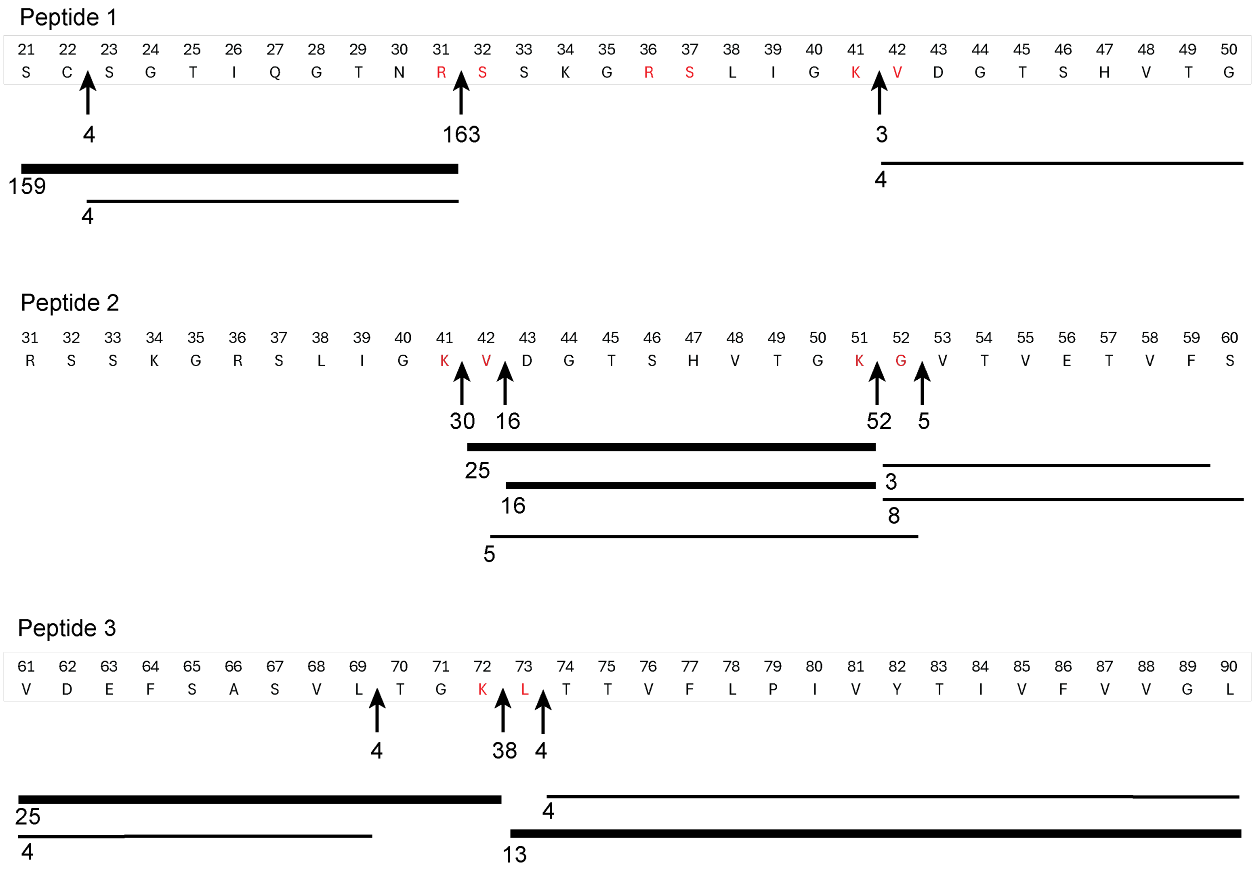

Supplementary Figure S5. Trypsin cleavage sites. Shown are the sequences of the three peptides synthesized for mass spectrometry analysis (position in the N-terminus and one letter amino acid code) and the span of the cleavage peptides with a Byonic score >300 and frequency ≧3 instances (indicated by lines with the number of instances at the lower left corner). Cleavage sites were tallied based on the span of the identified peptides. Note, sites generating small tryptic peptides are under-represented, as they are generally too small to be detected (*e.g.*, the cleavage peptides that would result from cleavage at the canonical site $R^{36}\downarrow S^{37}$ in Peptides 1 and 2). Cleavage efficiency for Peptides 1 and 2 was 100%. Amino acids labeled in red include the canonical site $R^{36}\downarrow S^{37}$ and the most frequent cleavage sites ($R^{31}\downarrow S^{32}$, $K^{41}\downarrow V^{42}$, $K^{51}\downarrow G^{52}$ and $K^{72}\downarrow L^{73}$).
